# The Association between Abnormal Long Noncoding RNA MALAT-1 Expression and Cancer Lymph Node Metastasis: A Meta-Analysis

**DOI:** 10.1155/2016/1823482

**Published:** 2016-01-27

**Authors:** Jun Wang, Yongsheng Pan, Jie Wu, Cheng Zhang, Yuan Huang, Ruizhe Zhao, Gong Cheng, Jinliang Liu, Chao Qin, Pengfei Shao, Lixin Hua, Zengjun Wang

**Affiliations:** State Key Laboratory of Reproductive Medicine, Department of Urology, First Affiliated Hospital of Nanjing Medical University, 300 Guangzhou Road, Nanjing 210029, China

## Abstract

Previous studies have investigated that the expression levels of MALAT-1 were higher in cancerous tissues than matched histologically normal tissues. And, to some extent, overexpression of MALAT-1 was inclined to lymph node metastasis. This meta-analysis collected all relevant articles and explored the association between MALAT-1 expression levels and lymph node metastasis. We searched PubMed, EmBase, Web of Science, Cochrane Library, and OVID to address the level of MALAT-1 expression in cancer cases and noncancerous controls (accessed February 2015). And 8 studies comprising 696 multiple cancer patients were included to assess this association. The odds ratio (OR) and its corresponding 95% confidence interval (CI) were calculated to assess the strength of the association using Stata 12.0 version software. The results revealed there was a significant difference in the incidence of lymph node metastasis between high MALAT-1 expression group and low MALAT-1 expression group (OR = 1.94, 95% CI 1.15–3.28, *P* = 0.013 random-effects model). Subgroup analysis indicated that MALAT-1 high expression had an unfavorable impact on lymph node metastasis in Chinese patients (OR = 1.87, 95% CI 1.01–2.46). This study demonstrated that the incidence of lymph node metastasis in patients detected with high MALAT-1 expression was higher than that in patients with low MALAT-1 expression in China.

## 1. Introduction

As is well known, malignant tumors are of great harm to human health and the trend of death is rising year by year. Cancer has already become a major cause of morbidity and mortality in most regions worldwide [1]. The 5-year survival rate remains low in many types of cancers, and numerous investigators are searching for biomarkers that may assist the diagnosis or prognosis of cancer [2]. In the tumor of the clinical pathological features, the occurrence of lymph node metastasis is a meaningful indicator for distant metastasis and survival in most cancers. Determination of lymph node metastasis is an important part of the TNM classification system, which dictates the choice of therapy and predicts prognosis of cancer patients. In the multistep process of metastasis, invasion into the lymphatic system has generally been believed as a key step of tumor cell dissemination [3]. The exact mechanism of metastasis through lymph nodes is unclear. Numerous studies have attempted to explain the process at the genetic level. Various kinds of genomic signatures have been reported to be associated with lymph node and distant metastasis [4–6]. However, most of the reports were only talking about a molecule specific to a particular tumor. A common molecular marker for predicting lymph node metastasis at the transcriptional level has not been demonstrated [7].

Recently, genome-wide transcriptome studies have confirmed that there are a large number of long noncoding RNAs (lncRNAs), which in the past had been dismissed as simply transcriptional “noise” [8]. LncRNAs are non-protein coding RNA molecules greater than 200 nucleotides in length. Diverse biological functions, including cell differentiation, development, and many disease processes, have been attributed to lncRNAs.

Metastasis-associated lung adenocarcinoma transcript-1 (MALAT-1), mapped to human chromosome 11q13, was originally identified as a prognostic marker for metastasis and patient survival in NSCLC [9]. Following studies have documented that MALAT-1 was aberrantly upregulated in multiple cancerous tissues and conferred proliferative and metastatic phenotypes to tumor cells [10, 11]. It promotes tumor growth and metastasis through different mechanisms, including recruiting SR family proteins [12], binding to the active regions of chromosome [13], and regulating alternative splicing of oncogenic mRNAs [14], depending on tissue contexts. High expression of MALAT-1 was associated with high-risk grade, metastasis, and poor prognosis of cancer patients [15–18]. It has been suggested that MALAT-1 expression may play a useful prediction role in the incidence of lymph node metastasis in some tumors. However, most studies examining the implications of MALAT-1 expression are limited by small sample size. Therefore, we conducted a systematic review and quantitative meta-analysis to clarify the exact diagnostic value of MALAT-1 expression association with lymph node metastasis in human cancers.

## 2. Methods

### 2.1. Literature Search

A literature search was conducted using the following electronic databases: PubMed, EmBase, Web of Science, Cochrane Library, and OVID. Besides, the following keywords and medical subheadings were used simultaneously in each set: “MALAT-1”, “lncRNA or long noncoding RNA”, and “cancer or carcinoma or tumor or neoplasms”. Alternative spellings were also considered. The search was completed on Feb 11th, 2015, and no lower date limit was used. All non-English articles were excluded and conference abstracts were not in the scope of our analysis because of the limited data indicated in them. The literature collected was performed by two independent researchers.

### 2.2. Inclusion and Exclusion Criteria

The articles collected were considered eligible if they met the following criteria: (1) they were published in English; (2) they measured MALAT-1 expression level in cancer patients; (3) they used tissue, saliva, or blood samples obtained from surgically resected cancer cases and noncancerous/normal controls from different patients for comparison; (4) related clinicopathologic parameters were included; (5) studies contained sufficient data for the computation of odds ratios (OR) and corresponding 95% confidence intervals (CI). Exclusion criteria were as follows: (1) letters, expert opinions, case reports, and reviews; (2) studies investigating the molecular structure and functions of MALAT-1; (3) studies without qualified data; (4) duplicates or continued work of previous publications; (5) being not yet published in English.

### 2.3. Data Extraction

**Figure 1:**
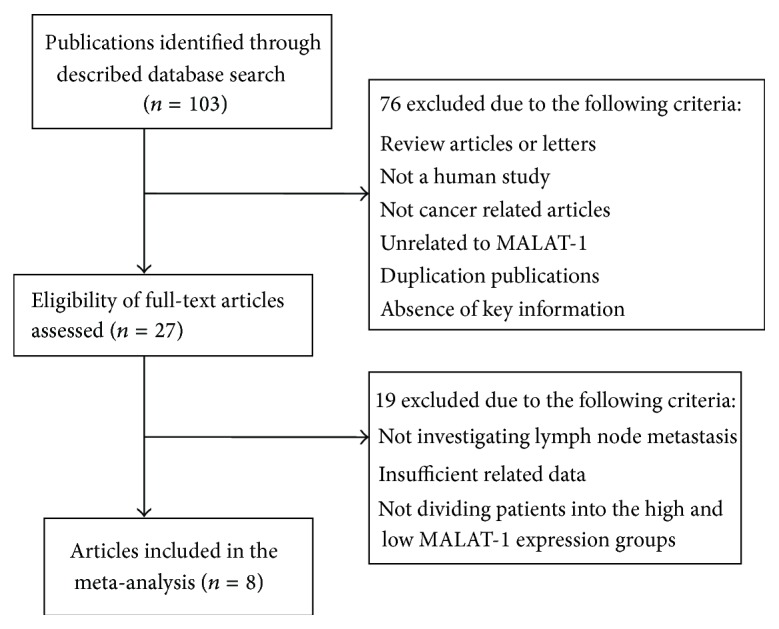
Flow chart depicting the study selection process.

Two investigators (Jun Wang and Yongsheng Pan) extracted data from the eligible studies independently, depending on the inclusion and exclusion criteria above. For disagreements, a consensus was achieved by a third investigator (Jun Wang). A flowchart describing the identifying process of qualifying studies is shown in Figure 1. Key components of a qualified study were recorded: first author, publication year, country of origin, ethnicity, tumor type, total number of patients, number of high MALAT-1 expression group and low MALAT-1 expression group, number of patients with lymph node metastasis in each group, and detection method of MALAT-1 expression levels.

### 2.4. Statistical Analysis

To determine the heterogeneity among the included studies, Higgins *I* ^2^ statistics were utilized. For *I* ^2^ statistics, an *I* ^2^ value greater than 50% was considered severe heterogeneity. The fixed effects model was adopted in the initial calculation of odds ratio with corresponding 95% CIs. If there was a significant statistical heterogeneity among the studies, the random-effects model was used for the analysis. Galbraith radial plot was conducted for individual studies to assess stability of the results. Sensitivity analysis was performed by sequential omission of individual studies to evaluate stability of the results. Moreover, we minimised the influence of heterogeneity by classifying the enrolled studies into subgroups built on similar characteristics. And potential publication bias was evaluated using a “funnel plot” and Begg's test. *P* value < 0.05 was considered statistically significant. By comparing the incidence of lymph node metastasis between high MALAT-1 expression group and low MALAT-1 expression group, we tried to ensure a thorough inquiry on the relationship between MALAT-1 expression levels and lymph node metastasis. We performed meta-analysis using Stata 12.0 version software.

## 3. Results

### 3.1. Study Selection and Characteristic

A total of 8 articles including a total of 696 patients were enrolled from a search of the above databases using the search strategy as described above [19–26] (Figure 1). The mean patient sample size was 87 (range 19 to 150). Six studies came from China and two articles from Japan. All the research objects were Asians. Among the eight studies, one focused on esophageal squamous cell carcinoma, two on renal cell carcinoma, one on colorectal cancer, one on osteosarcoma, two on pancreatic cancer, and one on gastric cancer. All cancerous specimens were well preserved before RNA extraction. No patients received chemotherapy or radiotherapy before surgery. All the diagnoses of lymph node metastasis were based on pathology. Among these patients, there are 360 with MALAT-1 upregulated (51.7%) and 336 with MALAT-1 downregulated (48.3%).

In the methods of lncRNA expression in cancer detection, quantitative real-time PCR (qRT-PCR) was the most common and effective method. In addition, microarray and in situ hybridization (ISH) were also used to measure the levels of lncRNA expression in cancerous tissues. In this study, qRT-PCR were utilized to divide high MALAT-1 expression group and low MALAT-1 expression group in all eligible articles. In previous study, qRT-PCR was utilized to detect the level of lncRNA expression in cancer, which included two ways: fluorescence probe (TaqMan) and fluorescence dye (SYBR Green). Of eight eligible studies in this meta-analysis, four used SYBR Green and four used TaqMan probe. Two kinds of methods were used to divide high MALAT-1 expression group and low MALAT-1 expression group: (1) the MALAT-1 expression levels were normalized to the glyceraldehyde-3-phosphate dehydrogenase (GAPDH) expression levels (high MALAT-1 group: MALAT-1/GAPDH ≥ 1.0; low MALAT-1 group: MALAT-1/GAPDH < 1.0); (2) the expression levels of MALAT-1 in cancerous and corresponding normal tissues (high MALAT-1 group: MALAT-1 expression level in cancerous tissue ≥ twofold the level in corresponding normal tissue; low MALAT-1 group: MALAT-1 expression level in cancerous < twofold the level in corresponding normal tissue). The clinical features of these eight included studies eligible for the meta-analysis are summarized in Table 1.

### 3.2. Study Results Report and Meta-Analysis

As indicated in Table 1, of 360 high MALAT-1 groups, 211 patients with different cancers had lymph node metastasis (58.6%). However, in low MALAT-1 group, there were only 105 patients with lymph node metastasis (31.3%). For this characteristic, it revealed that high expression levels of MALAT-1 may play an important role or promote the lymph node metastasis in patients with different systemic cancer.

**Figure 2:**
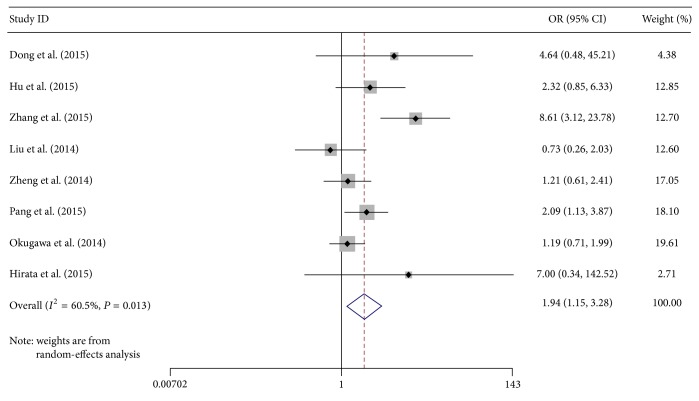
Forest plot for the association between MALAT-1 expression levels and lymph node metastasis by random-effects model. Squares and horizontal lines represent study-specific ORs and 95% CIs, respectively. The areas of the squares correspond to weights, and the diamonds represent the overall ORs and 95% CIs.

We performed an overall analysis of the data from studies containing abnormal expression of MALAT-1 and ORs from a variety of cancers. For high MALAT-1 expression group versus low MALAT-1 expression group, the studies were found to have moderate heterogeneity (*I* ^2^ = 60.5%, *P* = 0.013), so a random-effects model was applied to calculate a pooled OR and its 95% confidence interval (CI) (1.94, 95% CI: 1.15–3.28), which was statistically significant (Figure 2). Through comparing the incidence of lymph node metastasis between high MALAT-1 expression group and low MALAT-1 expression group, we found that there was a significant difference in the incidence of lymph node metastasis between the two groups. This result demonstrated that patients detected with high MALAT-1 expression in cancerous tissues may tend more to lymph node metastasis.

**Figure 3:**
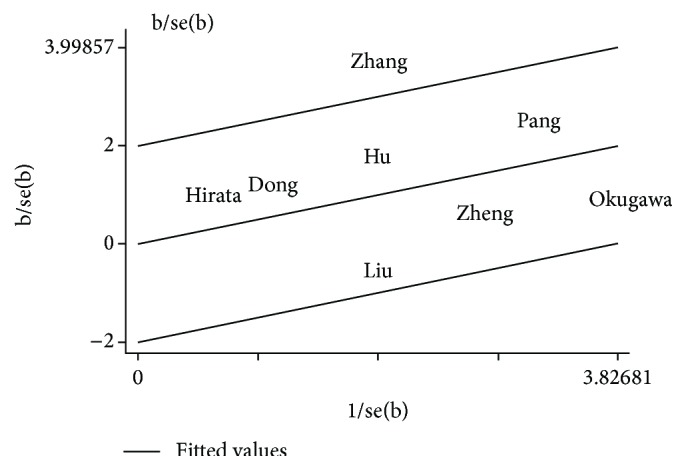
Galbraith radial plot for detecting the heterogeneity.

**Figure 4:**
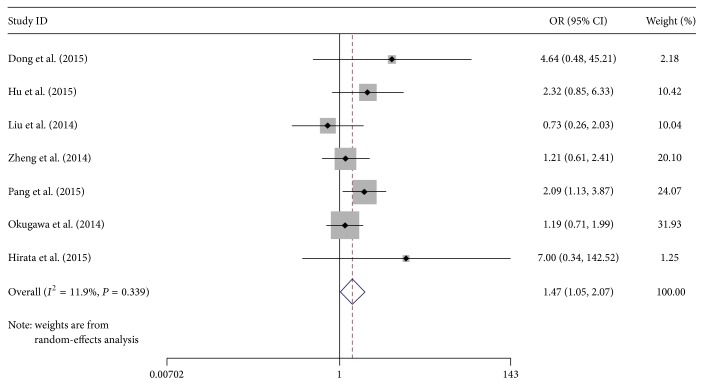
Forest plot for the association between MALAT-1 expression levels and lymph node metastasis (after removing one study).

Then, Galbraith radial plot was performed in order to show heterogeneity and its possible source more obviously. Related results as shown in Figure 3 demonstrated that one article (Zhang et al. [25]) had a significant influence on overall heterogeneity. Therefore, we removed this research to detect whether overall heterogeneity has changed. As we predicted, when high MALAT-1 expression group was compared with low MALAT-1 expression group, the studies were not found to have moderate heterogeneity (*I* ^2^ = 11.9%, *P* = 0.339), and pooled OR and its 95% confidence interval (CI) (1.48, 95% CI: 1.10–2.00) also had significance (Figure 4).

### 3.3. Subgroup Analysis

**Figure 5:**
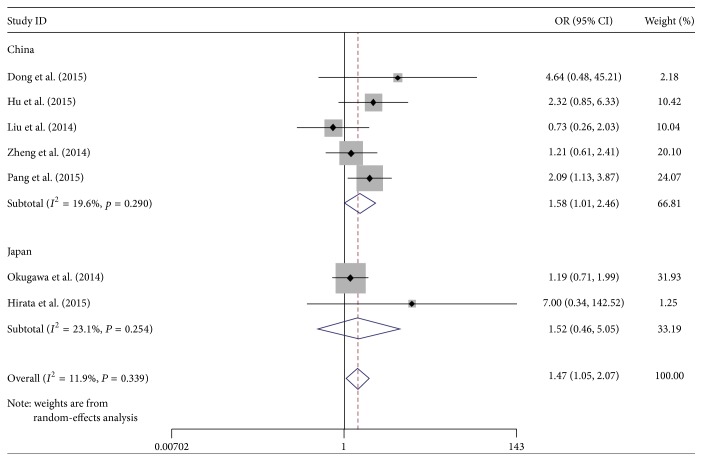
Forest plots of merged analyses of the association between MALAT-1 expression levels and lymph node metastasis in the different country subgroups (after removing one research).

**Figure 6:**
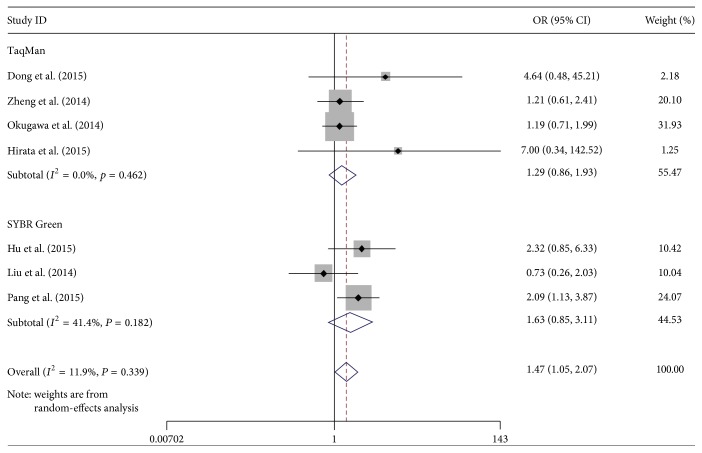
Forest plots of merged analyses of the association between MALAT-1 expression levels and lymph node metastasis in the different detection method subgroups (after removing one article).

We also performed a subgroup analysis by country and detection method. The results indicated a significant relation between MALAT-1 high expression and lymph node metastasis, which was also exhibited in China (OR: 1.58, 95% CI: 1.01–2.46). Subgroup analysis on other factors comprising detection method did not alter the significant incidence of lymph node metastasis impact of MALAT-1 high expression (Figures 5 and 6).

### 3.4. Assessment of Publication Bias and Sensitivity Analysis

**Figure 7:**
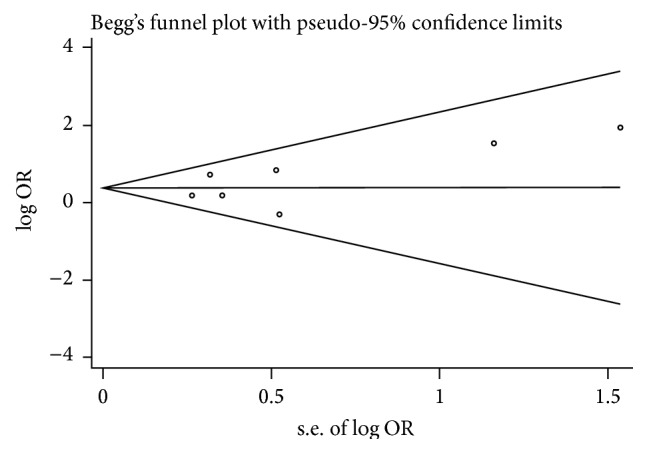
Begg's funnel plots of the publication bias for overall merged analysis (after removing one study). Each point represents a separate study.

**Figure 8:**
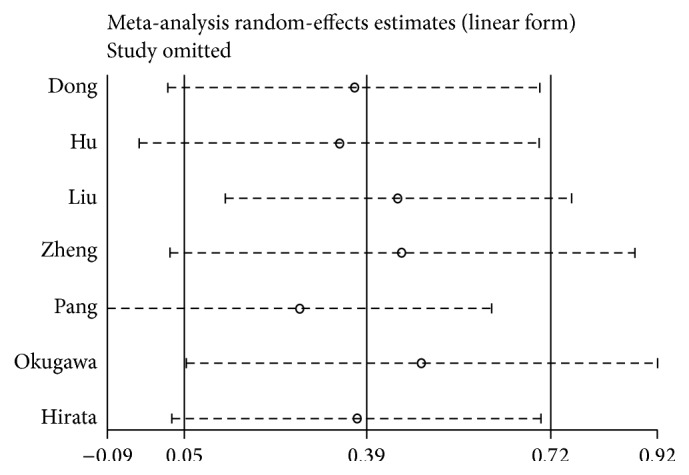
Sensitivity analysis of each included study except one.

To assess publication bias in this study, the included studies were conducted using funnel plots and Begg's test. As shown in Figure 7, Begg's funnel plots were almost symmetric. Thus, there was no evidence for significant publication bias in this meta-analysis. Meanwhile, one study (Zhang et al. [25]) was omitted to measure its effects on the pooled OR for deregulated MALAT-1 associated with lymph node metastasis in the sensitivity analysis. No individual study dominantly influenced overall OR, as presented in Figure 8.

## 4. Discussion

Metastasis-associated lung adenocarcinoma transcript-1 (MALAT-1)/nuclear enriched abundant transcript 2 (NEAT2) has been described as a regulator of metastasis and motility, and its expression is associated with metastasis in non-small cell lung cancer. It is a noncoding RNA of more than 8,000 nt derived from chromosome 11q13 [9]. MALAT-1 is overexpressed in multiple types of human malignancies, including hepatocellular cell carcinoma, lung adenocarcinoma, endometrial stromal sarcoma, and colorectal cancer [27–30].

Previous studies revealed that the expression levels of MALAT-1 in paired primary cancerous tissues exhibited higher than that in adjacent noncancerous tissues. Overexpression of MALAT-1 was associated with high-risk grade, metastasis, and poor prognosis of cancer patients [16–18, 30]. More importantly, scholars also have found that the expression levels of MALAT-1 can serve as a potential new therapeutic target and diagnostic urinary biomarker for prostate cancer [31]. In addition, knocking down MALAT-1 with targeted siRNAs could decrease the migration and invasion of tumor cells in vitro. On the contrary, enhanced expression of MALAT-1 could promote tumor cell matrigel invasion.

To our knowledge, lncRNAs regulate gene expression through various mechanisms, including transcription, posttranscriptional processing, chromatin modification, genomic imprinting, and protein function regulation [32, 33]. Two alternative models of action were proposed for MALAT1, including gene expression regulation and alternative splicing. Previous studies showed that MALAT-1 promotes cancer progression, mainly through the regulation of gene expression, for example, the epithelial mesenchymal transition associated genes, ZEB1, ZEB2, and Slug and the apoptosis related genes, CASP3, CASP8, BAX, BCL2, and BCL2L1 [34].

This meta-analysis aimed to examine the association between deregulated MALAT-1 expression and lymph node metastasis of multiple cancers. Our analysis combined the outcomes of 696 cancer patients from 8 individual studies, indicating that MALAT-1 high expression significantly predicted a high incidence of lymph node metastasis in cancer patients (OR 1.94, 95% CI 1.15–3.28).

In this systematic review, the test for heterogeneity of the included studies was significant. Primarily, we used a random-effects model during pooling data, but the model did not identify the source of heterogeneity. Moreover, Galbraith radial plot was used to clarify the possible source of heterogeneity in this study and reveled that one article (Zhang et al. [25]) might have a significant influence on overall heterogeneity. And when we excluded this study, the heterogeneity was disappearing (*I* ^2^ = 11.9%, *P* = 0.339).

Heterogeneity is a potential problem in interpreting the results of any meta-analysis. Through our in-depth analysis, we thought the potential source of heterogeneity for this paper might be as the following factors: (1) tumor specimens preservation methods were different after surgical resection. In this paper, tissues were collected in liquid nitrogen until use. (2) In included studies, qRT-PCR reaction condition and reaction system were different, the difference in this literature may affect the results. (3) Potential heterogeneity might relate to specific tumor clinical pathological parameters. In this study, clear cell renal cell carcinoma clinical pathological type may influence the overall results and is expected to result in heterogeneity. (4) Furthermore, due to the fact that an optimal threshold has not been defined, the cut-off defining cancer with MALAT-1 expression is arbitrary, which might produce heterogeneity. (5) cut-off value in this text might account for part of the interstudy heterogeneity.

As revealed in subgroup analysis, the incidence of lymph node metastasis in Chinese cancer patients had significance associated with MALAT-1 high expression (OR: 1.58, 95% CI: 1.01–2.46), while cancer patients in Japan show no significant result (OR: 1.52, 95% CI: 0.46–5.05). This diversity may be involved in race, environmental factors, medical level difference, and accessor methods, and so on. Subgroup analysis demonstrated that two kinds of detection method (TaqMan and SYBR Green) had no significant difference between deregulated MALAT-1 and lymph node metastasis. However, the total effects of the combined two methods were significant (OR: 1.47, 95% CI: 1.05–2.07) (Figure 6). This result may indicate that unified detection method for the division of MALAT-1 expression groups is important for this research.

Nevertheless, publication bias to be assessed and sensitivity analysis in this meta-analysis showed no significant results. We also thought our review has several limitations: (1) it is still necessary to conduct larger-size and better design studies to confirm our results; (2) the major limitation of this meta-analysis was that patients included in our study were all Asians; because of this, our finding may just represent patients from Asia; (3) literature reported tumor category is limited, so that we cannot actually confirm the MALAT-1 overexpression influence lymph node metastasis in whole human tumors; (4) a unified criterion for the division of MALAT-1 expression groups is important for this research.

To sum up, this study revealed that the incidence of lymph node metastasis in patients detected with high MALAT-1 expression was higher than that in patients with low MALAT-1 expression in China. The levels of MALAT-1 expression in cancers could become an independent and novel promising candidate for predicting disease staging and prognostic for human cancer patients.

## Figures and Tables

**Table 1:** Characteristics of individual studies included in the meta-analysis.

Year	Surname	Ethnicity	Country	Cancer type	Total number	MALAT-1 expression				Detection method
						High	High with LNM	Low	Low with LNM	
2015	Dong	Asian	China	Osteosarcoma	19	14	13	5	1	TaqMan
2015	Hu	Asian	China	ESCC	54	25	16	29	8	SYBR Green
2015	Zhang	Asian	China	RCC	106	46	33	60	5	SYBR Green
2014	Liu	Asian	China	PC	45	26	11	19	11	SYBR Green
2014	Zheng	Asian	China	CRC	146	73	23	73	19	TaqMan
2015	Pang	Asian	China	PC	126	63	46	63	22	SYBR Green
2014	Okugawa	Asian	Japan	GC	150	88	66	62	39	TaqMan
2015	Hirata	Asian	Japan	RCC	50	25	3	25	0	TaqMan

ESCC: esophageal squamous cell carcinoma; RCC: renal cell carcinoma; PC: pancreatic cancer; CRC: colorectal cancer; GC: gastric cancer.
